# Amorphous Phosphorus in Senile Arteriosclerosis

**Published:** 1914-01

**Authors:** 


Amorphous Phosphorus in Senile Arteriosclerosis. The author has used the red amorphous phosphorus in senile arteriosclerosis for several years. Given originally as a substitute for ordinary phosphorus in senile debility it was found that it was eliminated as amorphous phosphate of lime and that the lime elimination was thereby increased. Weil's experiments showed that the lime elimination in arteriosclerosis was diminished. Phosphorus has the property of combining with lime and increasing the lime assimilation. In the small doses which can be given when the ordinary phosphorus is employed the phosphorus will combine with the lime of the food and increase the amount of lime salts in the body. When given as amorphous phosphorus the dose is two grains or more several times a day, and with a lime-free diet the lime required for the combination necessary to secure the elimination of the phosphorus excess, is drawn from the abnormal lime deposits. This appears to be the rationale of the treatment and explains the good results obtained from its use. From *Diseases of Old Age,* by I. L. Nascher, M. D., to be published shortly by P. Blakiston's Son & Co., Philadelphia.

